# AI-Assisted Tuberculosis Detection and Classification from Chest X-Rays Using a Deep Learning Normalization-Free Network Model

**DOI:** 10.1155/2022/2399428

**Published:** 2022-10-03

**Authors:** Vasundhara Acharya, Gaurav Dhiman, Krishna Prakasha, Pranshu Bahadur, Ankit Choraria, Sushobhitha M, Sowjanya J, Srikanth Prabhu, Krishnaraj Chadaga, Wattana Viriyasitavat, Sandeep Kautish

**Affiliations:** ^1^Department of Computer Science and Engineering, Manipal Institute of Technology, Manipal Academy of Higher Education, Manipal 576104, India; ^2^Department of Electrical and Computer Engineering, Lebanese American University, Byblos, Lebanon; ^3^Department of Computer Science and Engineering, Graphic Era Deemed to be University, Dehradun 248002, Manipal, India; ^4^Department of Project Management, Universidad Internacional Iberoamericana, Campeche, C.P. 24560, Mexico; ^5^Department of Information Technology, Manipal Institute of Technology, Manipal Academy of Higher Education, Manipal, Karnataka, India; ^6^Department of Shalakya Tantra, Sri Dharmasthala Manjunatheshwara Institute of Ayurveda and Hospital, Bengaluru, India; ^7^Department of Kayachikitsa, Sri Dharmasthala Manjunatheshwara Institute of Ayurveda and Hospital, Bengaluru, India; ^8^Business Information Technlogy Division, Department of Statistics, Faculty of Commerce and Accountancy, Chulalongkorn University, Bangkok, Thailand; ^9^Janaki College for Professional Studies, Janakpur, Nepal

## Abstract

Tuberculosis (TB) is an airborne disease caused by *Mycobacterium tuberculosis*. It is imperative to detect cases of TB as early as possible because if left untreated, there is a 70% chance of a patient dying within 10 years. The necessity for supplementary tools has increased in mid to low-income countries due to the rise of automation in healthcare sectors. The already limited resources are being heavily allocated towards controlling other dangerous diseases. Modern digital radiography (DR) machines, used for screening chest X-rays of potential TB victims are very practical. Coupled with computer-aided detection (CAD) with the aid of artificial intelligence, radiologists working in this field can really help potential patients. In this study, progressive resizing is introduced for training models to perform automatic inference of TB using chest X-ray images. ImageNet fine-tuned Normalization-Free Networks (NFNets) are trained for classification and the Score-Cam algorithm is utilized to highlight the regions in the chest X-Rays for detailed inference on the diagnosis. The proposed method is engineered to provide accurate diagnostics for both binary and multiclass classification. The models trained with this method have achieved 96.91% accuracy, 99.38% AUC, 91.81% sensitivity, and 98.42% specificity on a multiclass classification dataset. Moreover, models have also achieved top-1 inference metrics of 96% accuracy and 98% AUC for binary classification. The results obtained demonstrate that the proposed method can be used as a secondary decision tool in a clinical setting for assisting radiologists.

## 1. Introduction

**Figure 1:**
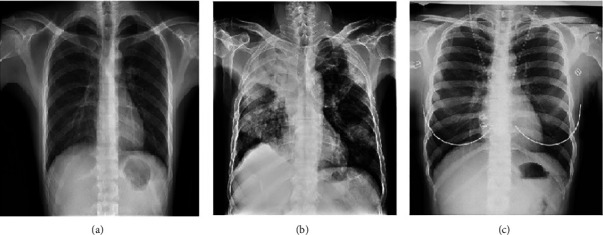
Illustration of Chest X-rays. (a) Healthy. (b) Viral infection. (c) Tuberculosis.

Lung diseases are often associated with excruciating pain and suffering as they affect the breathing pattern of the patient due to suffocation and related symptoms. Tuberculosis (TB) is one such detrimental variant of lung infections that has created a devastating impact on humankind. Tuberculosis is caused by the *Mycobacterium tuberculosis* bacteria. In general, the lungs are the main target area of this disease, but it can also affect other parts of the body. TB is a contagious disease, i.e., when people infected with TB cough or sneeze, they transmit the disease-causing bacteria in the air. Only a small quantity of these germs are enough to effectively infect a healthy person. Although scientific discoveries and research have been helping to curb the growing influence of TB, the meagre annual medical progress rate in this sector has been unsuccessful in bringing a drastic drop of TB affected patients. According to the Global Tuberculosis Report, 2020, generated by the WHO [1], approximately 10 million people were affected by TB worldwide in 2019. Additionally, HIV/AIDS and TB form a deadly combination. The HIV infection significantly minimizes the strength of the immunity system of an individual, which serves as a favourable condition for an HIV positive patient to contract TB. Out of the 1.4 million deaths caused by TB in 2019, more than 200 thousand patients were HIV positive. In Figure 1, description of chest X-ray of a healthy, viral, and tuberculosis patient is provided [2].

Manual inspection methods are labour intensive and require expertise in that particular domain to give accurate inferences. Therefore, there arises a need to merge the state-of-the-art technological advancements with medical theories and procedures. Artificial intelligence as a sector has boomed magnificently in recent decades and has spread across numerous industries. The entry of artificial intelligence into the medical field has propelled the progress rate tremendously in several types of research and has given scientists the freedom to explore uncharted territories.

Thus, the government authorities and multinational companies in the health sector have been encouraging institutions and academicians to utilize the maximum potential of machine learning and artificial intelligence to accelerate research in important domains such as medical imaging, diagnosis, and drug development. Dermatology, which is an image and screening intensive subfield of medicine, has great synergy with deep learning techniques pertaining to image processing [3–6]. Automation is carried out in electronic health records [7], therapeutic chatbots [8–10], and health monitoring.

Systems [11, 12] have rapidly expanded with the help of deep learning algorithms. With the help of natural language processing, scientists are able to identify and enhance the drug-drug interaction in medical literature [13]. Predictive modelling and decision making are important applications of AI in primary care [14–16].

In TB, computer-aided detection (CAD) is the most widely used artificial intelligence tool. The tool analyses the patient's chest X-rays and determines if the patient is affected by TB. This process reduces the load on the radiologists to meticulously scan through every radiographic film and ultimately speeds up the screening process.

The unique approach in this research is to leverage the power of normalization-free networks to escalate X-ray image classification. We experiment on different normalization-free architectures and compare them with their standard versions to prove their superiority. An AUC of 98% is obtained by the base line model. This proves the potential use of CAD in the diagnosis of the deadly TB.

### 1.1. Contributions and Related Literature

A number of papers have been published which diagnose TB using deep learning. However, in this article, the novelty lies in a variety of factors from data augmentation and regularization to the use of classification network. Chest Visualization is also discussed in this research which will help the medical personnel. Following are our vital contributions to this research.

1. The use of RandAugement algorithm for augmentation. It is an automated technique which is known to deliver accurate results.
2. The use of progressive resizing to augment the images based on their sizes.
3. Classification using normalization-free network instead of batch normalization to tackle performance issues.
4. Adaptive grading clipping technique used in this research to avoid the problem of exploding gradients.
5. Furthermore, the Score-CAM technique used for thorough chest visualization from a medical view-point.

A variety of algorithms have been used and compared with the proposed model. There is a drastic improvement in the results obtained by using the above preprocessing and classification techniques. A lot of public datasets have been tested too.  Table 1 gives information about the various datasets used in this research. The main novelty in the work is the usage of RandAugment for the augmentation of the data and the utilization of normalization-free network for the classification. Problems with batch normalization: batch normalization is a key component of most image classification models, but it has many undesirable properties. There can be discrepancies between the behavior of the network during training and testing times. While training, the network might have learned and trained to certain batches, which makes the network dependent on that batch-wise setup. So, it might not perform well when a single example is provided at inference. Batch normalization breaks the independence between examples within a batch. This means the examples selected in a batch are significant and lead us to two more prospects: Batch Size matters and inefficient Distributed training that will lead to the network cheating the loss function. To overcome these problems, normalization-free network is utilized and also, the comparison of NFNet with other state of the art image classification algorithms is mentioned in the paper.

Few recent studies have reported changes in X-ray images in patients at the onset of TB. Here, we review related literature which use AI and deep learning to diagnose this bacterial infection. Liu et al. [2] employed AlexNet and GoogleNet with shuffle sampling to classify TB from chest X-rays and achieved an accuracy of 85.68%. Hooda et al. [27] designed an ensemble of AlexNet, GoogleNet, and ResNet to detect TB. The models were trained from scratch and achieved an accuracy of 88.24%. The benchmark TB dataset (TBX11 K) was proposed by Liu et al. [26]. The authors compared the performance of various deep learning models to achieve simultaneous detection and classification with an accuracy of 88.2%. Hwang et al. [24] designed a modified AlexNet that was pretrained on ImageNet dataset to achieve TB classification. It achieved an accuracy of 90%. The pipeline data augmentation technique and ResNet18 model developed by Ghorakavi et al. [28] achieved an accuracy of 65.77181%. A D-CNN that involved demographic details along with images achieved better accuracy than I-CNN that considered only images in TB detection from chest X-rays [29]. Lakhani and Sundaram et al. [30] employed AlexNet and GoogleNet to detect TB. The ensemble of both gave an AUC score of 0.99. Nguyen et al. [31] proposed that ImageNet weights were insufficient for the modalities like X-Rays and discussed a new technique to obtain low level features by training the models in a multiclass multilabel scenario. Among the models trained, DenseNet-121 outperformed others by achieving an AUC score of 99% and 80% on Shenzhen and Montgomery datasets. Performance of pretrained AlexNet VGG16, VGG-19, and Xception ResNet 50 were compared in [32]. They identified that the features from shallow layers gave better results than deeper layers. Sivaramakrishnan et al. [25] employed InceptionV3 to obtain the classification of TB. They arrived at a conclusion that a supervised deep learning model trained from one population would not have the same diagnostic performance in another population. Hijazi et al. [33] proposed an ensemble of VGG16 and InceptionV3 which utilized the features extracted from original images of chest X-rays and canny edge detected images. The model with probability decision and variation of features led to improved TB detection. Pasa et al. [34] proposed a simple CNN model with five convolutional blocks to achieve the TB classification. They also employed saliency maps and grad-CAMs visualization techniques and discussed them from the radiologist's perspective. VGG16, Artificial Neural Network (ANN), and a customised CNN were employed to classify between drug resistant and nondrug resistant TB [35]. The ANN outperformed other models as the size of the dataset was small. Vajda et al. [36] employed the atlas-based lung segmentation and feature extraction to obtain the sets of features that could differentiate normal chest.

X-rays from the TB suspicious ones. A neural network-based classifier was utilized to achieve the classification. A maximum area under the curve and accuracy of 99% and 97.03% was obtained with Montgomery and the Shenzhen dataset. An ensemble of classifiers by combining the Support Vector Machines (SVMs) trained using the features extracted from chest X-Rays utilizing GoogLenet, ResNet, and VggNet is proposed in [37]. The models performed extremely well in diagonising TB. In research by Yan et al. [38], ML was used to diagnose TB using CT images.

892 CT scans of patients were included. The overall classification accuracy obtained was 81.09% to 91.05%. The paper concluded that deep learning has a lot of scope to diagnose TB in the future. Deep learning based Mycobacteria detection was conducted in [39]. Two autopsy patients and 40 biopsy cases were used for this research. A 100% specificity was obtained by the algorithms. The sensitivity ranged from 29% to 86%. Podder et al. [40] used transfer learning to diagnose COVID-19 and other diseases. The Modified Xception classifier obtained an accuracy of 84.82%.

The dataset contained chest x-rays of patients. Mondal et al. [41] used an optimized InceptionResNetV2 to diagnose COVID-19. The dataset contained both COVID-19 and non COVID-19 CT images. A maximum accuracy of 96.18% was obtained. A review of various ML and DL algorithms for COVID-19 diagnosis was conducted by Mondal et al. [42]. 52 articles were considered for this extensive review. Results concluded that ResNet-18 and DenseNet 169 were the efficient algorithms. Bharathi et al. [43] developed “CO-ResNet,” an optimized algorithm which diagnoses COVID-19 from chest X-rays. A maximum accuracy of 99% was obtained which distinguished COVID-19 from other viral diseases. Bharathi et al. [44] used deep learning to detect lung infections using chest x-rays. Among all the algorithms, VDSNet performed best with 73%. Bharati et al. [45] used CNN and lightGBM to identify the lung carcinoma. ResNet 50 architecture was compared with different models. The metrics used were log loss and ROC curve.

## 2. Materials and Methods

In this paper, we propose a three-fold method aimed as follows:

1. Detect if a chest X-ray is related to a healthy patient or to a patient infected with tuberculosis.
2. To discriminate between sick (but not TB) and TB.
3. To highlight the affected areas in the chest X-ray symptomatic of TB.

### 2.1. Dataset Description

The study is carried on two sets. The first set is henceforth referred to as “Set 1” and the second set is henceforth referred to as “Set 2.” The model's performance is tested by utilizing the two sets. The first set is referred as “Test Set 1” and the second set is referred as “Test set 2.”

Set 1: this set is comprised of the below datasets:

1. Tuberculosis X-ray (TBX11 K): the dataset [26] contains 11,200 chest X-rays from individual patients of different age groups and genders. Out of the 11,200 images, only 8400 images were provided with ground truths. Hence, they are considered for training and validation. This subset is split into 3800 (Healthy X-rays), 3800 (Sick but not TB X-rays), and 800 (TB X-rays). The images are in PNG format.
2. Montgomery: this dataset consists of images collected from the *tuberculosis* control program of the Department of Health and Human Services of Montgomery County, MD, USA. 80 X-Rays are normal and 58 X-Rays are abnormal with manifestations of tuberculosis. The images are in DICOM format.
3. Shenzhen: this dataset consists of X-ray collected by Shenzhen No. 3 Hospital in Shenzhen, Guangdong providence, China. The set consists of 326 normal X-Rays and 336 abnormal X-Rays [18]. The images are in JPEG format.
4. DatasetA + DatasetB: the images are collected from the National Institute of TB and respiratory diseases, New Delhi, India [19]. Dataset A includes 78 images for each class, showing normal lungs and others with various TB manifestations. Dataset B includes 75 images for each class, showing normal lungs and other TB manifestations.

The images are in DICOM format

- Test Set 1:
- A separate held out set is utilized. The test set is composed of 1200 Healthy images, 1200 Sick but not TB images, and 2800 images belonging to TB. The set is retained as an online challenge [26]. All the images are in PNG format.
- Set 2: this set is comprised of the following datasets:
- Belarus, NIAID, and RSNA : the Belarus set [46] includes 306 CXR images belonging to TB positive cases collected from the National Institute of Allergy and Infectious Diseases, Ministry of Health, Republic of Belarus.
- The NIAID Set [47] consists of 2800 TB positive images collected from seven different countries. The RSNA set [48] consists of 3,094 normal images collected from the RSNA pneumonia detection challenge.
- Test set 2:
- Training set 2 is split into a 60-20-20 ratio. This set has 700 images under the normal category and 700 images under the TB category.

#### 2.1.1. Augmentation Techniques and Regularization

To prevent the model from overfitting on the train test split, we utilized 4 layers of RandAugment [49] followed by conversion of the images to Grayscale (with 3 channels, where *r* = g = *b*) which prevented any colored X-rays after the RandAugments were done. RandAugment is an automated data augmentation method. The main goal of RandAugment is to remove the need for a separate search phase on a proxy task. The search space has two interpretable hyperparameters M and N. N represents the number of augmentation transformations to apply sequentially, and M denotes the magnitude for all the transformations. A number of works enumerated a policy of choosing which transformations to apply out of available transformations, and probabilities for applying each transformation. In this algorithm, to maintain image diversity and reduce the parameter space, learned policies and probabilities for applying each transformation are replaced with a parameter-free procedure of always selecting a transformation with uniform probability 1/K. K represents the number of transformation options. With only these two parameters, RandAugment can be utilized uniformly across different tasks and datasets. It matches or exceeds the performance of the other autoaugment techniques. In the proposed work, a constant value of 0.5 for “M” (magnitude hyper-parameter) is chosen.

**Figure 2:**
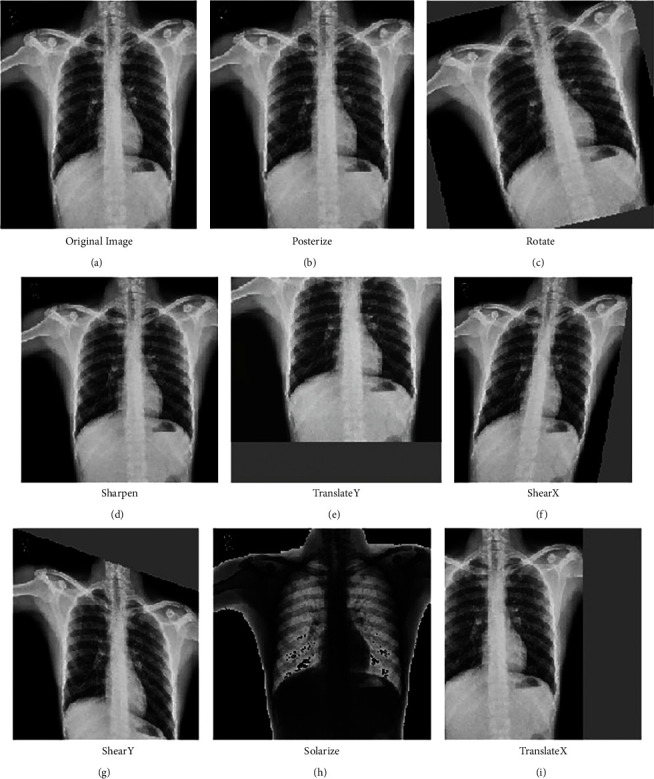
Different types of augmentations applied using RandAugment: (a) Original image, (b) Posterize, (c) Rotate, (d) Sharpen, (e) TranslateY, (f) ShearX, (g) ShearY, (h) Solarize, and (i) TranslateX.

Also, the augmentations are applied at every epoch, effectively giving our model new images to train/validate against at each epoch. Augmentations are also applied on the validation set due to the nature of random train/validation split. This resulted in the proposed model to perform better on the test set, than it did on the validation during training. These operations provided very strong explicit regularization. However, to further achieve better model generalization, more techniques are employed. In Figure 2, various augments chosen using RandAugment can be seen.

### 2.2. Progressive Resizing

The size of an image plays a crucial role in determining the model's performance. Images with smaller dimensions lead to a small network capacity and thus requires lesser regularization. On the other hand, images with larger dimensions require extensive computations and are more prone to be overfitted. Thus, when the model is being trained with variable image sizes, the strength of regularization should be adjusted accordingly to boost the model's accuracy and improve its performance i.e., an image with a smaller dimension works better with weaker regularization and weaker augmentation techniques. Similarly, images with larger dimensions work better with a stronger extent of regularization and augmentation techniques to defeat overfitting. The psuedocode for progressive resizing is described in Algorithm 1.

## 3. Classification Using Normalization-Free Network

Artificial Intelligence has impacted medical diagnosis in a positive way. The models have been deployed in various hospitals and medical facilities to assist the doctors and radiologists. It also offers a second opinion and stabilizes the conclusion derived from the X-ray images.

The batch normalization technique is used to scale the activations as they pass through the hidden layers and helps in restricting them to a certain range of values. This is achieved by inserting normalization layers after every hidden layer. Despite its several advantages, the batch norm was not the most appropriate alternative as it did not help us in achieving the best possible performance for our specific use case.

One issue always encountered with batch normalization enabled models is the performance discrepancy during training and testing. During the training process, the batch norm technique requires the model to train over numerous batches of preferably large size and the statistics (mean and variance) are computed corresponding to the minibatch.

This tends to make the model batch-dependent. Hence, when fewer images, lesser in comparison to the batch size, are put to test on the model, and the results produced are often inaccurate and deviate from the ground truth. For instance, the batch normalization-enabled model would be successful in training effectively over the X-ray images present in a large batch size of 128, but, when it would be put to test only on a single X-ray image, the statistics (mean and variance) of the test image might vary significantly from the minibatch statistics, which would ultimately lead to erroneous results.

Another issue faced while employing the batch normalization technique is the slower prediction time and extra computation. To tackle the problem of changing ranges of weights between the layers and stabilizing the learning process, the batch norm technique introduces a normalization operation after every hidden layer. Although the desirable results for the above problems are attained, the training time gets compromised. Hence, the normalizing operation after every hidden layer increases the model run-time as well as the computational resources employed by it.

For the reasons stated above, we needed to utilize a network that would not only provide us with robust training performance and highly accurate results but also be time efficient. The normalization-free network achieves significantly better performance than most of its competitors (e.g., EfficientNet-B7) by eliminating the use of batch normalization and slightly modifying the architecture of the normalization-free ResNets [50]. Apart from being lightweight and training efficiently on larger batch sizes, the NFNet utilizes residual branches and Adaptive Gradient Clipping (AGC) that bolster the model's performance [50].

1. Approach towards residual branches: this component of the NFNet architecture is its “normalization-free” feature. The residual branches of the NFNet architecture make use of 2 scalars, namely *α* and *β* [50]. These scalar quantities help in scaling the activations at the beginning and the end of the residual branch, thus restricting the activations to a certain range. This feature of the NFNet is analogous to the scaling operation done by the normalization layers inserted after every hidden layer in the batch normalization enabled models. Hence, the NFNet adopts this merit from the batch normalization technique to compensate for its absence.
2. Utilization of adaptive gradient clipping: during the backpropagation process, when the norm of the gradient gets bigger as it passes through each layer such that there is an exponential increase in its magnitude, the weights get updated inaccurately and consequently, and the network performance gets hampered. This is known as the exploding gradient issue and it is extremely critical to solving it to improve the overall performance of the model.

The gradient clipping technique is a popular option utilized to tackle exploding gradients. During the training process, when there is a certain norm of a gradient that is very high such that it surpasses a threshold value “lambda” (symbol needs to be inserted), the norm is said to be scaled to the threshold (formula associated with it to be added). Thus, the maximum possible value for a norm of a gradient is the threshold value lambda and any value above the threshold is clipped. For a gradient vector *G* = ∂L/∂*θ*, where *L* denotes the loss and denotes a vector with all model parameters, the standard clipping algorithm clips the gradient before updating as shown in (1).

(1) $$\begin{array}{c} G=\left\{\begin{array}{cc} \frac{XG}{\left\|G\right\|}\text{,} & \text{if }\left\|G\right\|>\lambda \text{,}\\ G\text{,} & \text{otherwise.} \end{array}\right. \end{array}$$

However, there are a few drawbacks associated with gradient clipping. The threshold value is very sensitive, and therefore, its appropriate selection is of paramount importance. Secondly, every high gradient jump which surpasses the threshold value is said to be clipped. But, while clipping, the weight associated with the corresponding gradient jump is not taken into account. There might be a few instances where the high gradient jump is justified by a significant corresponding weight, but, since the weight factor is not evaluated, all such gradients are also clipped.

As an improvisation to the existing gradient clipping technique, the adaptive gradient clipping (AGC) takes into account the weight associated with a particular gradient's norm during the filtering process. The AGC is computed as the ratio of the norm of the gradient to the norm of the weight associated with that particular layer. It measures how much the weights are affected for a single gradient descent step. For instance, if the value of the norm associated with a certain gradient is high enough such that it surpasses the threshold, but the weight associated with the same is significant, then the high jump is said to be justified and the gradient is not clipped. However, if the weight associated with the abnormal rise in the norm of the gradient is not sufficient, then that particular gradient is scaled to the threshold value and is said to be clipped. Hence, the weight corresponding to the particular norm of a gradient plays a crucial role in the clipping decision as shown in (2).

(2) $$\begin{array}{c} G_{i}^{l}⟶\left\{\begin{array}{cc} G_{i}^{l}⟶\lambda \frac{\left\|W_{i}^{l}\right\|*F}{\left\|G_{i}^{l}\right\|F}\text{,} & \text{if }\frac{\left\|G\right\|F}{\left\|W_{i}^{l}\right\|*F}>\lambda \text{,}\\ G_{i}^{l}\text{,} & \text{otherwise,} \end{array}\right. \end{array}$$

where *W*^*l*^ ∈ R^*N*×*M*^ denotes the weight matrix of the *l*^*th*^ layer, *G*^*l*^ ∈ R^*N*×*M*^ denotes the gradient with respect to *W*^*l*^, and the Frobenius norm is denoted by ||·||_*F*_.

**Figure 3:**
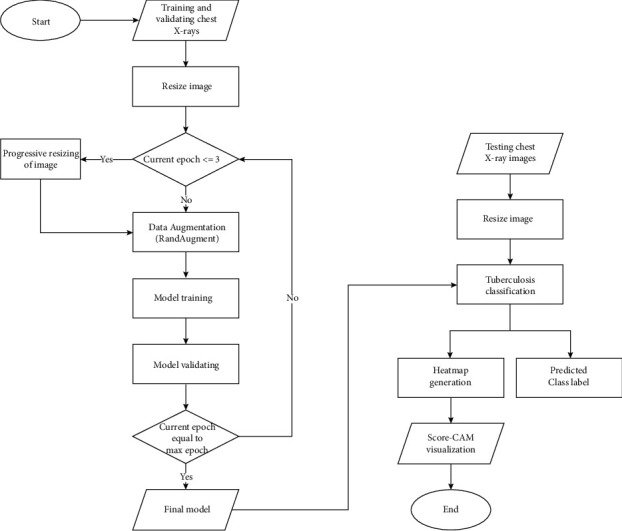
Flow of the proposed model.

**Figure 4:**
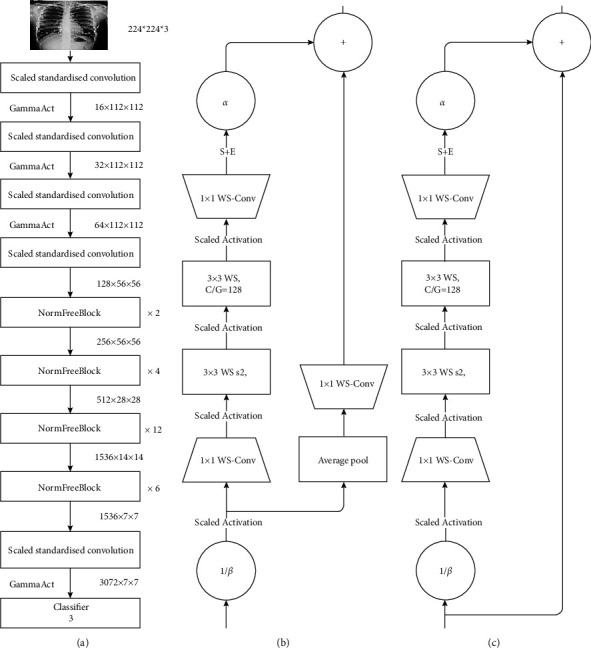
(a) Normalization-free network architecture. (b) NFNet transition block. (c) NFNet non transition block.

Additionally, the AGC serves to be advantageous for our specific use case because it significantly reduces the probability of exploding gradients occurring during the training process by preventing the imprecise updation of the weights. Accurate classification of X-ray images is of prime importance as it is the question of life and death for a particular patient; any slight error can lead to deleterious circumstances. Although batch normalization was not specifically designed to tackle the exploding gradient issue, it was able to mitigate its effects, but not eliminate it. AGC on the other hand was specifically devised to tackle exploding gradient issues and does a much better job in reducing the erroneous effects of gradient explosion as compared to batch norm [51]. The entire flowchart of the proposed model is described in Figure 3.  Figure 4 explains the architecture of various deep learning networks.

We denote h_*i*_ ∈ R^*m*×*n*^ to be the outputs of previous block and *α* = 0.2 be a constant scale factor.

Nontransitional block.

The matrix *X* ∈ ^*m*×*n*^, denotes the output of a nontransitional block.

*λ* is the composition of operations on *h*_*i*_ that when summed with the original input give the output *X* i.e.,

(3) $$\begin{array}{c} X=\lambda \left(hi\right)+hi\text{.} \end{array}$$

We can denote *λ* as follows: *λ* = fk ◦ fk−1 ◦··· ◦ f1(hi) where (*f*_*k*_: *k* = 1*…*11).

The composition of operations are as follows: (1) *f*_1_ = (1*/β*)*h*_*i*_. Here, input of previous block is scaled by a factor 1*/β.* Where *β*_*i*_ = ^p^*Var* (*h*_*i*_) and *Var* (*h*_*i*+1_) = *Var* (*h*_*i*_) + *α*^2^. *β* is the standard deviation of the inputs *h*_*i*_. (2) For *i* = 2...n − 2, if *i* is an even number: fi = ScaledAct (x) Otherwise: fi = *WSConv* (*x*).

The scaled activation function [50, 52] (the Gamma Activation) is a ReLU activation scaled by a constant factor $r=\sqrt{2/1-\left(1/\pi \right)}$.

WSConv [52] is the standard weight standardization function: *w*_*ij*_⟶*w*_*ij*_′.

This is done to reparameterize original weights $w_{ij}^{\prime }⟶w_{ij}-u_{i}/\sqrt{N\sigma_{I}}$,

where *ui* = (1*/N*)P*j Wij* and *σ*2 = (1*/N*)P*j*(*Wij* − *ui*)2. (3) f10 = SE(x), which is the queeze and Execitation block. (4) f11 = *α*x, (scalar-vector multiplication), where *α* (as defined earlier) is a scalar value that regulates the rate at which the variance of the activation increases.

Transitional block.

The transitional block is almost identical to the previously described in nontransitional block, with a few changes.

Here, instead of summing outputs of a series of operations *λ*(*h*_*i*_) with the previous layers input directly, the series of functions is as follows:

(4) $$\begin{array}{c} g\left(h_{i}\right)=WS\text{Conv ◦AvgPool◦ ScaledAct}\left(\left(\frac{1}{\beta }\right)\times h_{i}\right)\text{,} \end{array}$$

where after scaling the inputs by (1*/β*) feeding the product into the scaled activation (Gamma activation) function.

The matrix is first reduced by average pooling and then expanded again by standard weight standardization.

The output of the transitional block is *Y* ∈ ^*m*×*n*^ = *λ*(*h*_*i*_) + *G*(*h*_*i*_).

**Figure 5:**
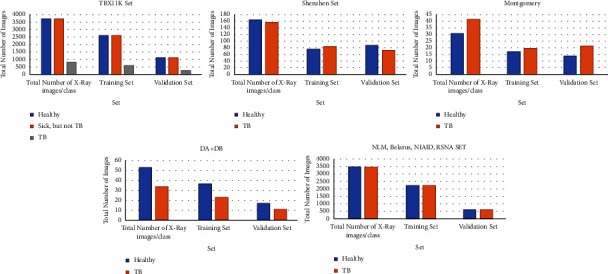
Dataset split.

The details of the training and validation datasets are described in Table 2 and figuratively given in Figure 5. The various training parameters for the NFNet model are explained in Table 3.

## 4. Performance Criteria for Classification Using Normalization-Free Network

The performance of different models for testing dataset was evaluated after the completion of training and validation phase and was compared using the following performance metrics: Accuracy, Sensitivity, Specificity, Area under 255 Curve (AUC), Average Precision, and Average Recall. TP represents true positive cases, TN represents true negative cases, FN represents false negative cases, and FP represents the number of false positive cases. The metrics are defined below:

(1) Accuracy: the number of positive and negative TB cases identified correctly among all classified cases. It is calculated using the equation given below:
  (5) $$\begin{array}{c} \text{Accuracy}=\frac{\left(TP+TN\right)}{TP+FN+FP+TN}\text{.} \end{array}$$
(2) Sensitivity: it is the number of positive TB cases identified accurately. It is also called recall. When the number of false negative cases are minimum, the sensitivity is extremely high. It is described using the formula given below:
  (6) $$\begin{array}{c} \text{Sensitivity}=\frac{\left(TP\right)}{TP+FN}\text{.} \end{array}$$
(3) Specificity: it is the number of negative TB cases identified correctly. When the number of false positives are minimum, the obtained specificity will be maximum. It is calculated using the formula given below:
  (7) $$\begin{array}{c} \text{Specificity}=\frac{\left(TN\right)}{FP+TN}\text{.} \end{array}$$
(4) AUC (Area Under Curve): the area under the curve when the graph is plotted between the true positive rate and false positive rate. When the AUC is high, it means the model is classifying the instances correctly.
(5) Average precision: it summarizes all the values of the precision-recall curve into a value. It also represents the mean of all the precision's obtained. It is calculated using the formula:
  (8) $$\begin{array}{c} {\text{Average}}_{\text{Precision}}=\left(\frac{\sum_{n}P_{n}}{n}\right)\text{.} \end{array}$$
(6) Average Recall: the mean of recalls calculated from thresholds of 0.5 to 1 to summarize the distribution. It is calculated using the formula:
  (9) $$\begin{array}{c} {\text{Average}}_{\text{Recall}}=\left(\frac{\sum_{n}R_{n}}{n}\right)\text{.} \end{array}$$

### 4.1. Visualization Technique: Score-CAM

To highlight the areas in the chest X-ray symptomatic of TB, we utilize the Score-CAM [53] technique.

The Score-CAM visualization technique, built on top of the CAM-based visualization method, follows a perturbation 265-based approach. This technique comprises two stages; the first stage passes the input images into a CNN and generates the activation maps. Subsequently, the maps are upsampled as they are smaller in dimensions as compared to the input image. In the second stage, the activation maps generated are pointwise multiplied with the input image and normalised. The normalization process significantly improves the discriminative ability of the model. The masked inputs are fed to CNN and corresponding scores of the specified target class are generated. This process is repeated 270 until it has been applied to all the generated maps. The Score-CAM metric is given as

(10) $$\begin{array}{c} L_{\text{score}-\text{CAM}}^{C}=ReLU\left(\underset{k}{\sum }\alpha_{k}^{c}A_{l}^{k}\right)\text{,} \end{array}$$

where

(11) $$\begin{array}{c} \alpha_{k}^{c}=C\left(A_{l}^{k}\right). \end{array}$$

Here, C(.) denotes the channel wise increase in confidence for a particular activation map. The ReLU function is used to eliminate those features which have had no impact on the target class. The proposed work utilizes Score-CAM as it does not require mask sampling or any process for optimisation. Gradients have not been utilized in the course of heat map generation. The removal of global average pooling layer (used in the class activation mapping technique) eliminates the need to retrain the entire process or make any changes to the network structure.

## 5. Experimental Results and Analysis

In this section, we evaluate the models on the parameters discussed in the previous section. The Score-CAM visualization is also explored in depth. Furthermore, the results are also classified using other deep learning models for TBX11k and Kaggle dataset.

### 5.1. TB Classification

**Figure 6:**
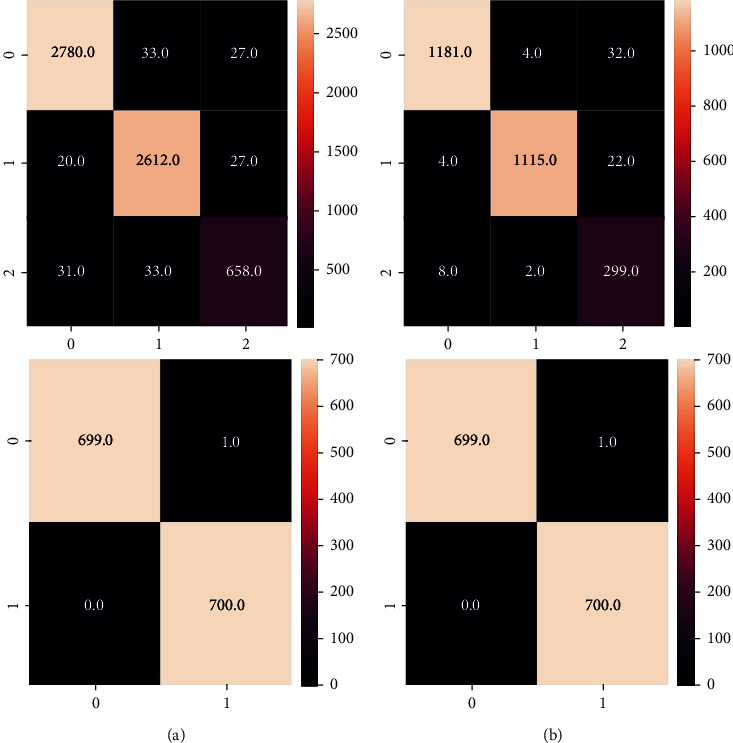
(a) Confusion matrices for the training dataset. (b) Confusion matrices for the validation dataset.

TB classification using the proposed normalization-free-network model is the main objective of this research.  Figure 6 describes the confusion matrix obtained by the two datasets (training + validation).  Figure 6(a) represents the confusion matrices for the training data. The matrix on the top represents the TBX11K dataset which consist of three classes namely: healthy, infection (but not TB), and TB. The bottom matrix is of the Kaggle dataset Test dataset 285 (2) which consists of healthy and TB images. These confusion matrices are obtained after training the normalization-free network model.  Figure 6(b) represents the confusion matrices of the above datasets, but on the validation set. As we can see, the false positive and false negative values are extremely low (NonDiagonal elements). This classifier promises us a good accuracy. All the metrics such as accuracy, precision, recall, and others can be effectively calculated using the confusion matrix.

**Figure 7:**
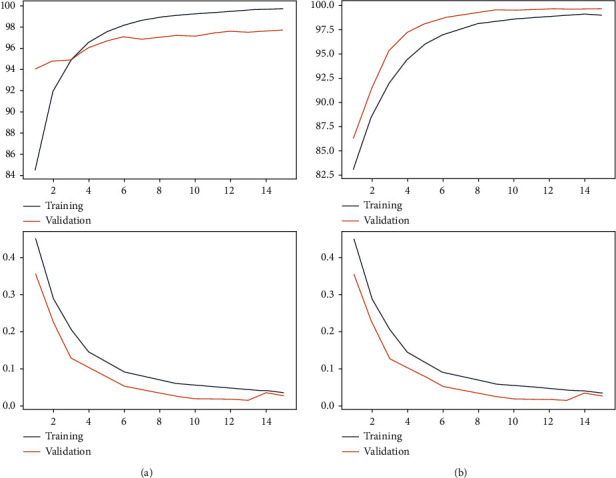
(a) Training and validation accuracy and losses versus Epoch for Set 1. (b) Training and validation accuracy and losses versus Epoch for Set 2.

Figure 7 represents the accuracies and losses against the number of epochs for the training and validation sets. From the plots, it can be observed that there is no overfitting since the accuracies and losses are almost similar between the training and test datasets. Further various deep learning architectures were used to classify the datasets along with our proposed model.

**Figure 8:**
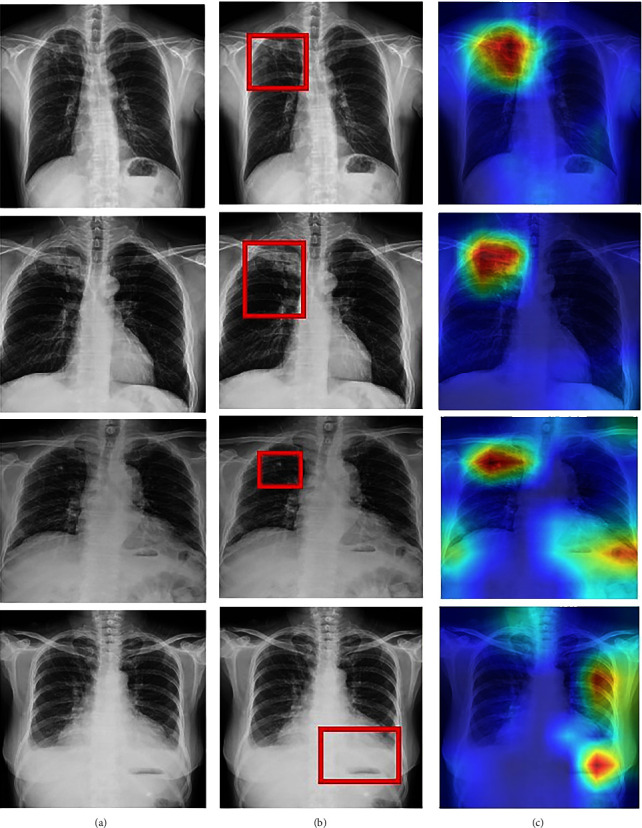
Score-CAM visualization of correctly classified TB (first two rows), infected ,and sick but not TB (last two rows) chest X-ray: (a) Original X-ray. (b) Ground truth given by domain expert. (c) Score-CAM heat map generated by the model.

**Figure 9:**
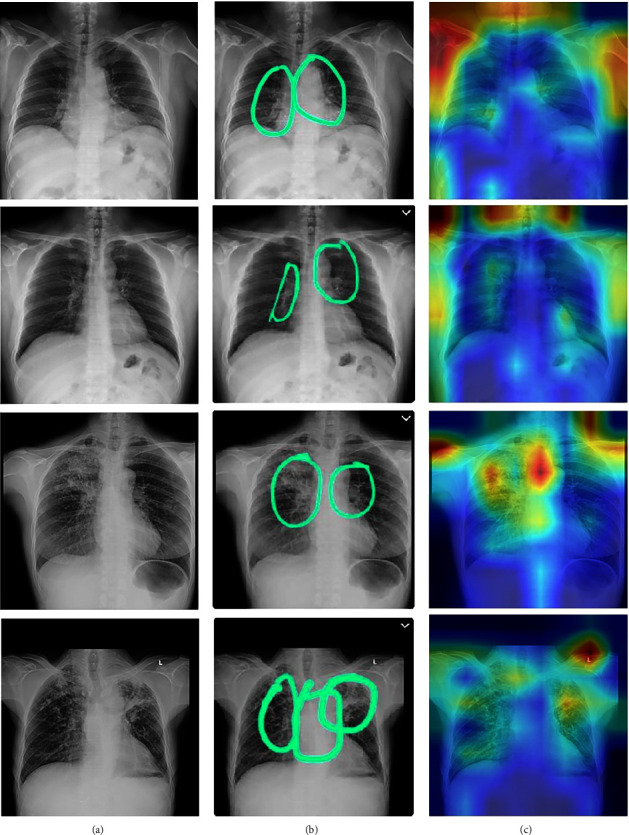
Score-CAM visualization of incorrectly classified actual sick classified as TB (first two rows) and actual TB classified as sick but not TB (last two rows) chest X-ray: (a) Original X-ray. (b) Ground truth given by domain expert. (c) Score-CAM heat map generated by the model.

The Score-CAM technique was utilized to highlight the regions of the lungs affected by TB. This chest visualization-295 technique can be used to highlight the abnormalities in the specific region in the lungs, aiding the doctor to understand the region of interest. As discussed in the previous section, this process consists of two stages. CNN is used in the beginning to generate activation maps. Furthermore, these activation maps are multiplied with the initial image using normalization. The normalization process is extremely important to discriminate between the various image classes. These modified images are again sent to the model for classification. All the generated maps are subjected to 300 of this procedure for accurate analysis.  Figure 8 shows the visual analysis of the chest X-ray. The first two rows represent the X-rays of a TB-infected patient. The last two rows are the X-rays of patients infected with other lung diseases (not TB). The first column represents the initial X-ray. The doctors have examined these x-rays and have labelled the region of interest (labelling). This is represented in the second column. The third column represents the marking of the region of interest by the Score-CAM algorithm. From the figure, it is observed that the algorithm identifies the region of interest accurately using a heat map (matches the ground truth given by the domain experts.) However, in some conditions, the heat maps generated can be wrong. This is depicted using Figure 9. The first two rows represent TB cases. The last two rows represent other lung diseases (not TB). The ground truths (labels) by the doctors are present in the second column. From the figure, it is clearly inferred that the generated heat maps are not the same as the ground truths (wrong classification). The Score-CAM is an effective algorithm, but some false positive and false 310 negative cases are observed.

The data was split into training and testing in the ratio of 80:20. The performance of the models are given in Tables 4 and 5. ResNet-18 is a highly efficient deep learning network which consists of 18 different layers. Millions of images can be easily loaded in this network. It can also classify images into a variety of classes. Furthermore, it is already trained on the ImageNet data. This model obtained an accuracy, AUC, sensitivity, specificity, average 315 precision, and average recall of 91.01%, 95.69%, 76.82%, 96.08%, 89.48%, and 88.75%, respectively, on the TBX11k dataset. For the Kaggle dataset, the obtained accuracy, AUC, sensitivity, specificity, average precision, and average recall are 90%,94%,72%,95%,87%, and 86.25%, respectively. ResNet50 is a deep residual network which is 50 layers deep. The network is stacked upon each other just like other artificial neural networks. This neural network is pretrained on the ImageNet dataset. The accuracy, AUC, sensitivity, specificity, average precision, and average recall on the TBX11k dataset were 98.76%, 87.02%, 96.99%, 93.67%, and 93.6%, respectively. For the Kaggle dataset, the obtained accuracy, AUC, sensitivity, specificity, average precision, and average recall were 92.61%, 97.6%, 84.02%, 96.88%, 92.41%, and 92.1%, respectively.

The DenseNet-121 is known to have 120 convolutional layers and four average pooling layers. The weights are spread over multiple inputs for optimal accuracy. These networks were specifically created to counter the vanishing gradient problem. The model obtained an accuracy, AUC, sensitivity, specificity, average precision, and average recall of 98.0%, 94.82%, 71.41%, 95.89%, 86.85%, and 85.13%, respectively. For the Kaggle dataset, the accuracy, AUC, sensitivity, specificity, average precision, and average recall obtained were 88.0%, 94.82%, 71.41%, 95.89%,86.85%, and 85.13% respectively. Another important CNN model is the DenseNet-201 which consists of 201 layers. It has been already trained by the ImageNet database and can effectively predict upto 1000 classes. The accuracy, AUC, sensitivity, 330 average precision, and average recall were 92.64%, 97.23%, 79.91%, 97.18%, 91.64%, and 90.6%, respectively, for the TBX11k multiclassification dataset. For the Kaggle dataset, the obtained accuracy, AUC, sensitivity, specificity, average precision, and average recall were 90.11%, 95.14%, 79.81%, 94.21%, 91.22%, and 80.45%, respectively.

**Figure 10:**
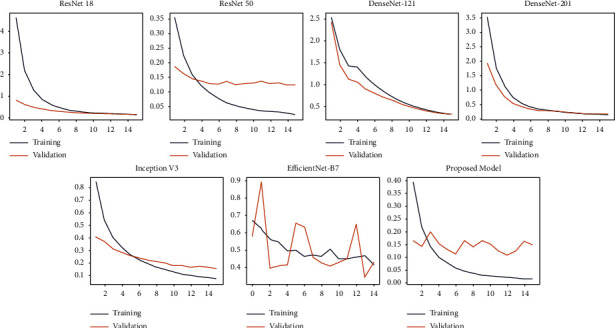
Training and validation losses versus Epoch of chest X-ray images present in TBX11k set.

**Figure 11:**
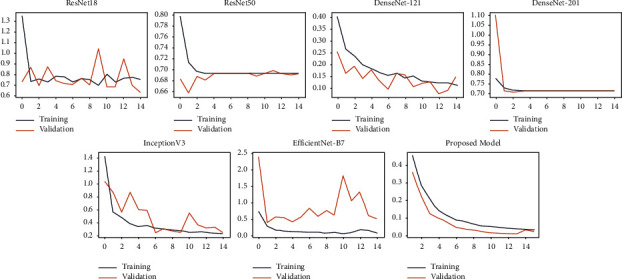
Training and validation losses versus Epoch of chest X-ray images present in Kaggle set.

InceptionV3 is a deep learning model used to classify images at a high accuracy rate. It includes both symmetric and asymmetric building neurons, including convolutions layer, max pooling, average pooling, drop outs, and fully connected layers. Softmax algorithm is used to compute the model loss. In this network, batch normalization is applied to the activation inputs. The InceptionV3 was able to obtain an accuracy, AUC, sensitivity, specificity, average precision, and average recall of 89.58%, 94.95%, 69.4%, 97.54%, 89%, and 86.21%, respectively. For the Kaggle dataset, the accuracy, AUC, sensitivity, specificity, average precision, and average recall obtained were 88.62%, 91.25%, 65.21%, 94.31%, 86.28%, and 84.77%, respectively. EfficientNet B7 is a rethinking scaling CNN. It returns an image classification model using transfer learning. This algorithm was developed by a company named AutoML. It also uses a compound coefficient to uniformly scale all the dimensions to its resolution. The obtained accuracy, AUC, sensitivity, specificity, average precision, and average recall were 84.07%, 73.82%, 51.78%, 95.86%, 82.14%, and 78.39%, respectively, for the TBX11k dataset. For the Kaggle dataset, the obtained accuracy, AUC, sensitivity, specificity, average precision, and average recall are 83.21%, 74.65%, 84.32%, 93.21%, 81.31%, and 75.45%, respectively. The proposed normalization-free network was able to perform better than all the models. The accuracy, sensitivity, specificity, average precision, and average recall obtained by the base models were 96.91%, 99.38%, 91.81%, 98.42%, 96.33%, and 96.1%, respectively. This proves that the model is highly efficient in classifying TB. For the binary classification dataset (Kaggle), the obtained accuracy, AUC, sensitivity, specificity, average precision, and average recall are 95.91%, 98.32%,91.78%, 91.67%, 95.33%, and 91.1%.  Figure 10 and Figure 11 describe the losses obtained during training and validation for the two datasets.

An outline of comparison with previous literature-based approaches on the TB datasets is compared. The performance is compared with regards to the following measures: accuracy, AUC (TB), sensitivity, specificity, average precision ,and average recall. In research by Li et al. [26], CNN was used to diagnose TB using chest X-rays. AlexNet and GoogleNet were the models used. An accuracy of 85.08% was obtained by the classifiers. Hooda et al. [27] used three architectures: ResNet, GoogleNet, and AlexNet to diagnose TB. The models were further ensembled together to obtain an accuracy and AUC of 88.24% and 93%, respectively. In an article by Liu et al. [26], usage of image-based classifiers for TB diagnosis was considered. The accuracy, sensitivity, specificity, and AUC are 88.2%,88.4%, 89.5% and 93.8%, respectively. Furthermore, TB screening was performed in [23] using deep learning based on chest X-ray images. Three datasets were considered and the AUC obtained by them were 96%, 93%, and 88%, respectively. The TBNet model was created in [28] which diagnosed TB using deep learning models. Mirroring, rotation, and other augmentation techniques were used in the research. However, the ResNet architecture was only able to deliver an accuracy of 81%. Our best model was able to obtain an accuracy, sensitivity, and specificity of 97%, 92%, 99% and respectively. The performance of the comparative researches is described in Table 5.

Tuberculosis is a dangerous disease and it must be identified early to prevent the onset of severe symptoms. AI can be used to diagnose tuberculosis at a faster rate. These models can further help the doctors and radiologists to come to a decision. The classifiers have a tremendous potential in healthcare fields in the near future (Table 6).

## 6. Conclusion

Tuberculosis is a dangerous bacterial disease which affects the lungs in the human body. It is very important to diagnose this infection early since appropriate treatments can be provided. Computer Aided Diagnosis is a trending topic in Medical Artificial Intelligence. In this research, deep learning-assisted TB diagnosis is performed using normalizer-free network. For augmentation, RandAugment was used to convert the images to gray scale. Further, progressive resizing is used to perform automated preprocessing. Adaptive grading clipping is used to tackle the problem of exploding gradients in this research. A variety of models have been tested and our models achieved an accuracy, AUC, sensitivity, specificity, average precision, and average recall of 98%, 99%, 92%,99%,97%, and 96.1%, respectively. Additionally, a technique called the Score-CAM was used to draw inference from the chest X-rays. Further, the model was compared with the other state-of-art research studies to prove its novelty and superiority. The classifiers can be extremely useful in healthcare and will assist the doctors and medical professionals in performing accurate diagnosis.

In the future, General Adversarial Networks can be utilized to handle data imbalance. This will further boost the efficiency of the model. Chest CT Scan images can be collected from hospitals for computer-aided diagnosis in the near future. The models should be tested on other TB datasets to prove its efficacy. This will make the models more trustworthy. The system can be made user friendly and medical staff can use the models to aid the diagnostic procedure. The deep learning models can also be used to diagnose other diseases such as COVID-19, malaria, liver disease, isochronic heart disease, chronic kidney disease, cancers, and others.

## Figures and Tables

**Algorithm 1:**
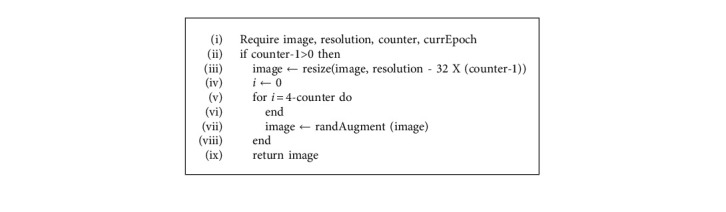
Progressive resizing.

**Table 1:** Tuberculosis: chest X-ray datasets.

Sr. No	Dataset name/source	Number of images	Reference
1	Find and treat screening program	TB:˙87, healthy: 113	[17]
2	TB-NEAT research study	TB: 66, Healthy: 134	[17]
3	Montgomery county chest X-ray set	TB:58, Healthy: 80	[18]
4	Shenzhen dataset	TB: 336, healthy: 326	[18]
5	DA	TB: 78, healthy: 78	[19]
6	DB	TB:75, healthy: 75	[19]
7	Belarus tuberculosis portal dataset	TB:304	[20]
8	Tuberculosis (TB) chest X-ray database	TB:3500, healthy: 3500	[21]
9	NIAID TB dataset	TB:3000	[22]
10	JSRT	TB: 154, healthy: 93	[23]
11	KIT	TB: 3828, healthy: 7020	[24]
12	ChestX-ray8	TB: 51,760, healthy: 60,360	[25]
13	TBX11 K	TB: 800, Healthy:3800, Sick: 3800	[26]

**Table 2:** Details of training and validation set for the classification problem.

Dataset	Type	Total no. of X-Ray images per class	Training set	Validation set
TBX11 K	Healthy	3800	2660	1140
	Sick but not TB	3800	2660	1140
	TB	800	560	240

Shenzhen	Healthy	167	116	51
	TB	160	112	48

Montgomery	Healthy	31	21	10
	TB	42	29	13

DA + DB	Healthy	54	37	17
	TB	34	23	11

NLM (shenzhen and montgomery), Belarus, NIAID, SNA	Healthy	3500	2100	700
	TB	3500	2100	700

**Table 3:** Training parameter of model for classification.

Parameter	Value used for training set 1	Value used for training set 2
Batch size	128	32
Learning rate	0.1	0.001
Epochs	15	15
Adaptive gradient clipping	0.16	0.16
Weight decay	1.00E-05	1.00E-05
Optimizer	Stochastic gradient descent	Stochastic gradient descent
StepLR scheduler	Every 2.4 steps	Every 2.4 steps
Gamma	0.97	0.97

**Table 4:** Performance metrics on TBX11K.

Model	Accuracy	AUC (TB)	Sensitivity	Specificity	Average precision	Average recall
ResNet18	91.01	95.69	76.82	96.08	89.48	88.75
ResNet50	94.85	98.76	87.02	96.99	93.67	93.6
DenseNet121	88.04	94.82	71.41	95.89	86.85	85.13
DenseNet201	92.64	97.23	79.91	97.18	91.63	90.6
InceptionV3	89.58	94.95	69.4	97.51	89	86.21
EfficientNet-B7	84.07	73.82	51.78	95.86	82.14	78.39
Proposed model	96.91	99.38	91.81	98.42	96.33	96.1

**Table 5:** Performance metrics on Kaggle dataset.

Model	Accuracy	AUC (TB)	Sensitivity	Specificity	Average precision	Average recall
ResNet18	90.23	94.31	72.21	95.41	87.54	86.25
ResNet50	92.61	97.6	84.12	96.88	92.41	92.1
DenseNet121	84.04	93.21	75.43	93.22	84.31	82.11
DenseNet201	90.15	95.26	79.86	94.21	91.22	88.45
InceptionV3	88.62	91.25	80.32	94.31	86.28	84.79
EfficientNet-B7	83.21	74.65	84.32	93.21	80.7	85.22
Proposed model	95.91	98.32	91.78	91.67	95.87	91.90

**Table 6:** Comparison of various research studies along with our proposed model.

References	Images	Model	Sensitivity	Specificity	AUC score	Accuracy
Liu et al. [2]	4701	Alex net and Google net	—	—	—	85.68
Hooda et al. [27]	1133	Alex net and Google net and ResNet ensemble	88.42	88	93	88.24
Liu et al. [26]	11200	SSD without pretraining and VGG net 1–16 as back bone	88.4	89.5	93.8	88.2
Hwang et al. [24]	10848	Modified pretrained Alex net	—	—	96.4	90
Ghorakavi et al. [28]	800	Reset 18 with data augmentation	—	—	—	65.771
Heo et al. [29]	10000	D-CNN	81.5	96.2	92	—
Lakhani and Sundaram [30]	1007	Ensemble of Alex net and Google Net	97.3	94.7	99	96
Nguyen et al. [31]	800	DenseNet-121	—	—	—	80
Sivaramakrishnan et al. [32]	112,782	Inception v3	72	82	70.54	99
Hijazi et al. [33]	800	VGG 16 and inception V3	90.91	88.64	—	89.77
Pasa et al. [34]	1104	CNN	—	—	92.5	86.2
Jaeger et al. [35]	135	ANN,CNN, VGG16	—	—	66	—
Vajda et al. [36]	814	Neural network	—	—	99	97.03
Lopes et al. [37]	1120	Gooogle net Res Net VGG net SVM	—	—	92.6	84.7
Proposed Model	3500	NF net model	91.81	98.42	99.38	96.91

## Data Availability

The data used to support the findings of this study are available from author Krishnaraj Chadaga upon request (krishnarajchadaga18@gmail.com).
